# Integration of pharmacogenetic data in epic genomic module drives clinical decision support alerts

**DOI:** 10.3389/fphar.2024.1458095

**Published:** 2024-12-20

**Authors:** Kimberly J. Newsom, Bradley Hall, Katherine A. Martinez, Scott Nelson, Petr Starostik, Khoa Nguyen

**Affiliations:** ^1^ Department of Pathology, Immunology and Laboratory Medicine, University of Florida, Gainesville, FL, United States; ^2^ Department of Pharmacotherapy and Translational Research, College of Pharmacy, University of Florida, Gainesville, FL, United States; ^3^ UF Health Information Services, Gainesville, FL, United States

**Keywords:** precision medicine, pharmacogenetics, electronic health records (EHR), clinical decision support systems, genomics, health informatics, health level seven (HL7)

## Abstract

**Introduction:**

The Precision Medicine Program (PMP) at the University of Florida (UF) focuses on advancing pharmacogenomics (PGx) to improve patient care.

**Methods:**

The UF PMP, in collaboration with the UF Health Pathology Laboratory (UFHPL), utilized Health Level Seven (HL7) standards to integrate PGx data into Epic’s Genomic Module to enhance the management and utilization of PGx data in clinical practice.

**Results:**

A key feature of the Genomic Module is the introduction of genomic indicators—innovative tools that flag actionable genetic information directly within the electronic health record (EHR). These indicators enable the effective presentation of phenotypic information and, when leveraged with existing clinical decision support (CDS) alerts, help provide timely and informed therapeutic decisions based on genomic data.

**Discussion:**

This advancement represents a significant shift in the utilization of genetic data, moving beyond traditional PDF reports to provide a comprehensive understanding of PGx data. Ultimately, this integration empowers healthcare providers with genomics-guided recommendations, enhancing precision and personalization in patient care, contributing significantly to the advancement of personalized medicine.

## 1 Introduction

The integration of genomic information into healthcare systems can revolutionize patient care by providing personalized insights based on an individual’s genetic makeup (Manolio et al., 2013). Since 2011, the University of Florida has been at the forefront of this revolution through its PMP, with a particular focus on PGx (Weitzel et al., 2014; Cavallari et al., 2017). The UFHPL conducts the majority of PGx tests internally, offering both single gene tests (e.g., *CYP2C19, CYP2D6, TPMT, NUDT15*) and a comprehensive panel, GatorPGx, which analyzes eight genes (*CYP2C19, CYP2D6, CYP2C9, CYP3A5, CYP4F2, CYP2C* cluster*, SLCO1B1, VKORC1*) crucial for drug metabolism and response.

A key principle of UF’s PMP has been the incorporation of health information technology into the EHR including the development of CDS tools to guide prescribing decisions based on drug-gene interactions (Lemke et al., 2023). Initially, these PGx-CDS tools were limited to providing interruptive alerts when relevant medications were ordered. While Epic has long supported CDS tools, genomic indicators are a key innovation introduced with the Genomic Module to enhance how genomic data is handled within the EHR. It introduces a new master file, the VAR (variant) database, designed to accommodate various types of genetic variants, including single nucleotide polymorphisms (SNPs), insertions/deletions (indels), and structural variants. This expanded capacity allows for more comprehensive storage, retrieval, and analysis of genomic data. Building on the enhanced data management capabilities of the VAR database, Epic also introduced genomic indicators within the Genomics Module to further improve the accessibility and interpretation of this information. The PGx genomic indicators offer several valuable features, including interpretation language tailored for both providers and patients, potential drug-gene interaction warnings with actionable recommendations, and a link to corresponding lab results. The Genomic Module can link genomic indicators to decision support tools that guide physicians through complex clinical choices. For example, if a patient has a genetic variant affecting drug metabolism, PGx indicators may be used as triggering criteria to alert clinicians helping them to avoid adverse drug reactions or select more effective therapies. Recognizing the need for more sophisticated genomic data management, UF Health decided in 2021 to implement Epic’s Genomic Module.

The integration of such complex genomic data presents unique challenges, particularly regarding data entry and interpretation. To address these challenges, UF utilized health informatics to develop a solution that streamlines PGx data interpretation and generates standardized information in HL7 format, compatible with the genomic module. HL7 is an international set of standards for transferring clinical and administrative data between healthcare software applications, ensuring consistent and structured data exchange while reducing the risk of misinterpretation or data loss. The standard HL7 message format consists of a message header obtained from the EHR. This header incorporates various components such as MSH (Message Header), PID (Patient Identification), ORC (Common Order), OBR (Observation/Order Request), OBX (Observation/Result), and NTE (Notes and Comments). Each of these components plays a specific role in organizing and conveying information within the HL7 message structure. The implementation of HL7 messaging in genomic data integration provides several benefits. It supports the inclusion of complex genomic data elements, such as variant annotations and clinical interpretations, while significantly reducing the time required for laboratory resulting and minimizing the risk of manual errors (Williams et al., 2019; Ayatollahi et al., 2019).

The overall objective of the project was to integrate Epic’s Genomic Module to enhance the management of PGx information in post-analytical therapeutic decisions; however, the complexity of integration warrants specific attention. The focus here is on the technical aspects of this integration, detailing the process of EHR resulting through HL7 integration and the implementation of downstream CDS tools.

## 2 Methods

The UFHPL uses Epic Systems Corporation (Verona, WI, United States) EHR with beaker laboratory information system (LIS). Epic Systems provides different modules tailored to specific laboratory disciplines, including Beaker Clinical Pathology (Beaker CP) and Beaker Anatomic Pathology (Beaker AP). At UFHPL, molecular resulting is done in the Beaker CP module, which allows data to be filed in discrete searchable data fields. The following describes the implementation of PGx data into Epic’s Genomics Module (November 2021 version), which facilitates the integration of complex genetic data into the EHR, enhancing the ability to utilize discrete genomic indicators for CDS.

To implement the Genomics Module, a custom middleware solution was developed to integrate PGx data with Beaker CP, utilizing a pre-existing Health Insurance Portability and Accountability Act (HIPAA)-compliant infrastructure. This infrastructure is designed around a Linux server that securely connects to Epic Bridges/NextGen Connect and a communal network drive (Chamala et al., 2020; Dolin et al., 2023). The system acts as an electronic intermediary, safeguarding the privacy and accuracy of clinical data while facilitating information exchange between the local network folder and Epic Bridges/NextGen Connect. Epic Bridges facilitates the integration of HL7 messages into the Epic EHR by using the Epic integration engine to validate incoming message values against expected ones.

A key challenge addressed during the integration was streamlining the conversion of PGx data into the correct nomenclature for entry into the EHR as discrete data, since this process can be complex and time-intensive when performed manually. For each gene, a genotype (expressed as a star allele) and a predicted phenotype are reported, and in some instances, an activity score may also be reported (Caudle et al., 2017). The predicted phenotype, based on the star allele nomenclature established by the Clinical Pharmacogenetics Implementation Consortium (CPIC), describes the individual’s metabolizer status for a given gene as Ultrarapid, Normal, Intermediate, or Poor (Caudle et al., 2017). Translating raw genomic data into star allele nomenclature typically requires an additional step, often involving third-party software, such as Allele Typer™ (Life Technologies), to ensure accurate genotype interpretation, particularly for complex genes like *CYP2D6* (Twesigomwe et al., 2020).

A workflow was created to bypass third-party software by implementing a Python-based solution to streamline the translation of raw data directly from the instrument. Python is used to process information in the form of CSV files. This flexibility allows for adjustments to assay content when necessary. The QS_Translator.csv file is used by the Python script to translate assay data into calls of Normal, Heterozygous, or Mutant, which allowed for easy management of nucleotide order combinations and simplified the addition of new assays. Following this step, the PGX_Translator.csv file, which contains all of the Probe Information, Assay IDs and all possible genotype calls, is used to convert the results into genotype calls. The script then returns the phenotype calls based on data in the GT_PT_Translator.csv file, which includes all relevant reportable information such as phenotype, activity score, comments, and whether the result should be flagged as abnormal in the EHR. Expertise from the UF Health PMP was utilized to ensure that the phenotype terminology aligns with the currently accepted CPIC guidelines (Caudle et al., 2017).

Institutions can access all files at https://github.com/UF-Health-Molecular-Pathology/PGX_HL7 and modifications can be made for institution-specific assays. The QS_Translator.csv file should be updated with assay-specific probe information. The PGX_Translator.csv file should be modified for assay-specific genotype calls and the GT_PT_Translator.csv file should be updated for assay-specific reportable information. In the Python code, the gene_symbol_replacements function should be updated to reflect assay specific genes, and the append_OBX_segments function should be updated to incorporate institution-specific mapping related to Epic Long Read Report (LRR) and Variant (VAR) coding.

**FIGURE 1:**
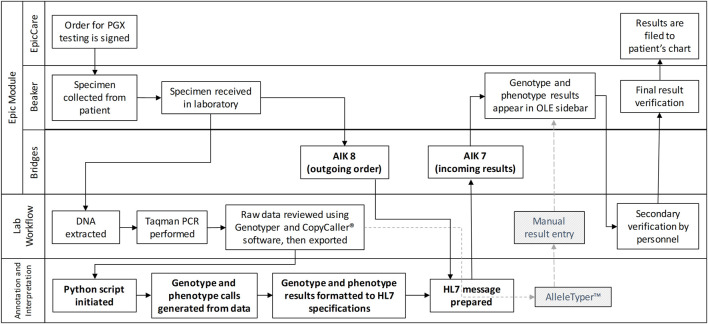
A Rummler-Brache diagram of the PGx workflow. Bolded items represent the new automated workflow utilizing the custom middleware solution. Items with dotted lines and hatched greyed out boxes represent the manual workflow. All other items are common to both workflows.

The overall infrastructure developed is shown in Figure 1 and illustrates the PGx workflow, with bolded elements highlighting the newly implemented automated workflow facilitated by the custom middleware solution. Components indicated by dotted lines and hatched greyed-out boxes represent the previously used manual workflow, while unaltered items are common to both processes. The sequence begins with orders being placed and signed in Epic, followed by the collection and laboratory testing of patient samples. In the automated workflow, upon receiving the sample in the laboratory, an outgoing ancillary order (Ancillary Interface Kind 8 [AIK8]) is transmitted to Epic Bridges, incorporating patient and order information to ensure accurate result filing. Subsequently, DNA extraction and TaqMan PCR are executed in the laboratory, and the data undergoes review before being exported for annotation and interpretation. In the manual workflow (depicted by a grey dotted line), data is uploaded to Allele Typer™ (Life Technologies) for annotation and interpretation, with results printed and manually entered into Beaker for review. In the automated workflow update, the in-house developed python script processes the data, generating genotype and phenotype calls. The results are formatted to HL7 specifications, matched with the AIK8 ancillary order to create an AIK7 result message and then automatically filed to Beaker using NextGen Connect and the Epic Bridges interface. Both workflows converge with a final results review, ensuring accuracy before verification and subsequent filing to the patient chart.

The development of the genomic indicators was a collaborative effort between the PMP, UFHPL, and the UF IT team. The PMP was responsible for reviewing CPIC guidelines to ensure consistency and accuracy, while also developing the interpretation language for both providers and patients for each gene. UFHPL generated the VAR records, which provide the structured genetic data necessary for the genomic indicators. The UF IT team mapped the genomic indicators to the VAR database using Epic’s PGx Turbocharger package, which includes a comprehensive suite of CDS, translation logic, genomic indicators, provider-facing clinical descriptions, and medication interactions. While the package supports all CPIC Level A guidelines, the implementation focused on a subset of these guidelines, and also made significant modifications to the translation logic. The translation logic in Epic consists of two components: (1) mapping tables that link genotypes to their corresponding phenotypes, which were tailored to include only the SNPs tested by UFHPL; and (2) rules for each genomic indicator that reference the mapping tables to determine when the indicator should be applied to a patient’s chart. For example, if the *CYP2C19* result maps to an intermediate metabolizer (IM), the corresponding indicator is added to the patient’s profile. The translation logic was expanded to apply to pre-existing results entered prior to the implementation via LRR components only. A utility to retroactively process those results was used to add indicators to patient charts. In collaboration with PMP, the logic for certain variants was also refined where the evidence did not fully align with current CPIC guidelines. Additionally, the decision support system was updated to check genomic indicators instead of traditional result components, and legacy CDS were revised to link to the smart text used in the genomic indicators, ensuring consistency in both clinician- and patient-facing content. The incorporation of these indicators now activates PGx-CDS, streamlining the update and monitoring processes for personalized medicine within the EHR system.

## 3 Results

**FIGURE 2:**
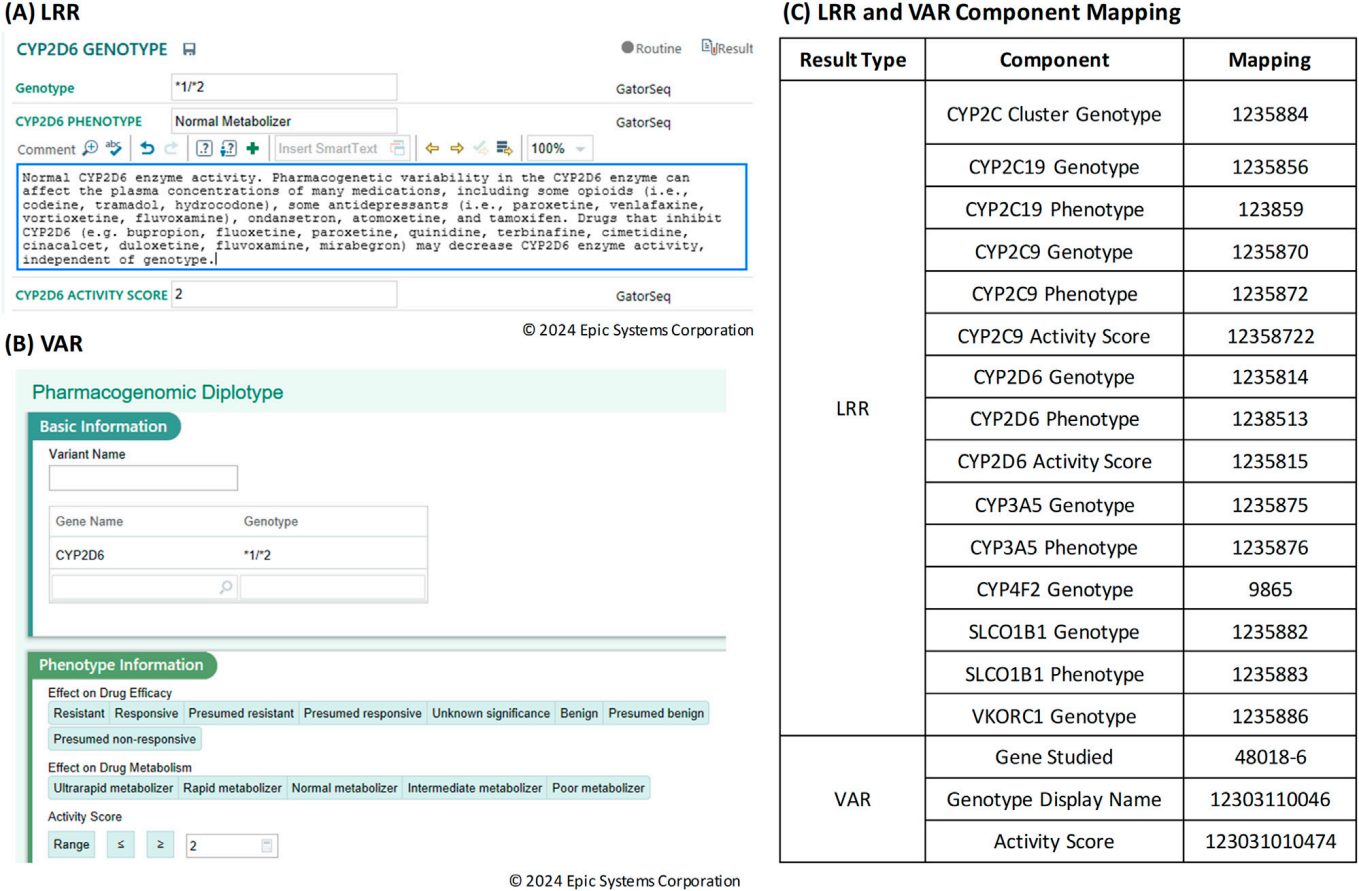
**(A)** Result entry screen in Beaker Clinical Pathology (CP) for Long Read Report (LRR) result types. **(B)** Result entry screen in Beaker Genomics Module for Variant (VAR) result types. **(C)** Table of Long Read Report (LRR) and Variant (VAR) component mapping.

In the transition to integrating the Genomics Module into a clinical workflow, it is important to understand the distinct data storage frameworks for CP and the Genomic Module within the Epic universe. CP results are stored in the LRR database, where data is saved as discrete records. Each of these components requires an individual record in the LRR, which makes it quite granular but also results in a larger number of records for each test. Figure 2A shows the result entry for *CYP2D6* where the result component is specific for each gene and result combination (e.g., *CYP2D6* Genotype, *CYP2D6* phenotype, *CYP2D6* Activity score). In contrast, results in the Genomic Module are stored in the VAR (variant) database. VAR uses a single set of records for each result type, simplifying the data storage and retrieval process. Figure 2B shows the Genomic Module result entry where the result components are not specifically tied to particular gene, so a new record is not needed for every gene/result combination. The table in Figure 2C shows the individual result components required for LRR vs. VAR for the same gene panel.

**FIGURE 3:**
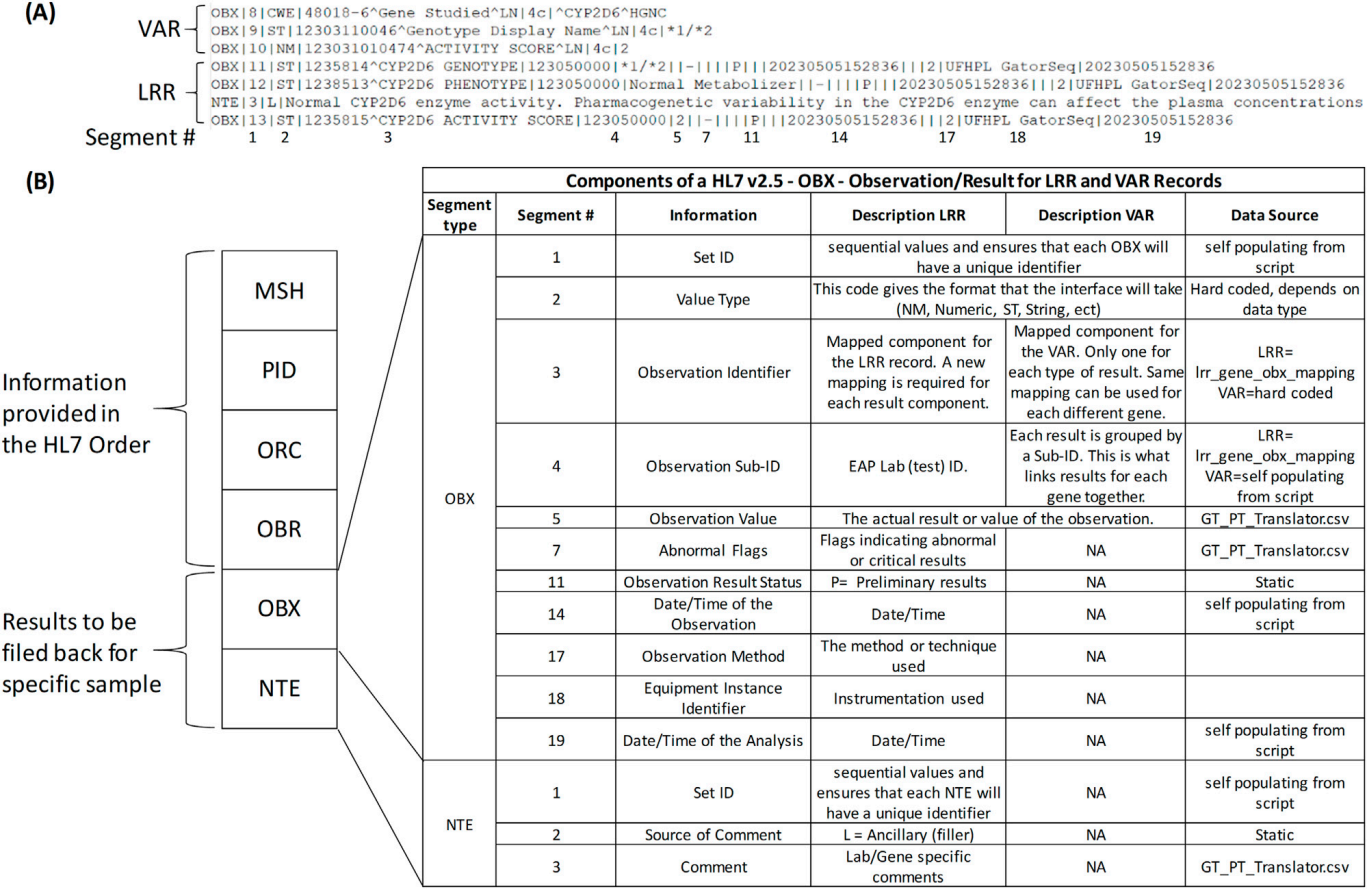
HL7 Messaging for Genomic Data Transmission. **(A)** Excerpt of a Health Level 7 (HL7) message for *CYP2D6*, including both Variant (VAR) and Long Read Report (LRR) result components represented as Observation (OBX) and Notes (NTE) segments. The segments are organized into specific fields and field numbers, separated by the pipe (|) symbol, with numbered segments corresponding to those shown in **(B)** at the bottom. **(B)** Detailed overview of the Observation (OBX) and Notes (NTE) segments within the HL7 message, focusing on the components for LRR and VAR records. This panel also highlights the data source from the Python script used to generate the HL7 message.

In the initial implementation of the Genomics Module, both LRR and VAR data types were included in the results to address key usability considerations. While the VAR view in the Genomics Module offers a comprehensive genomic perspective, it lacks integration within the results review tab, where LRR results are easily accessible. To ensure continuity for clinicians and enhance the overall review experience, both VAR and LRR were included to provide a more complete and convenient presentation of genomic results. To support the transmission of both data types, HL7 messaging was used. Figure 3A shows an excerpt of an HL7 message for *CYP2D6*, containing both VAR and LRR result components (OBX/NTE segments). The OBX and NTE segments are broken down into specific fields and field numbers, separated by the pipe (|) symbol. Figure 3B provides a detailed overview of the components within the HL7 message, specifically focusing on the OBX and NTE segments for LRR and VAR records. To generate the HL7 message, a Python script is utilized to populate each component, ensuring accurate data representation. For instance, in OBX segment 1 (OBX-1), the Set ID guarantees a unique identifier for each OBX, and this is self-populated from within the script. The Value Type (OBX-2) is a hard-coded value that indicates the format of the interface based on the data type. The Observation Identifier (OBX-3) is mapped for the LRR record, requiring new mappings for each result component. In contrast, for VAR, only one mapping is utilized per result type, which can apply to different genes. The Observation Sub-ID (OBX-4) represents the EAP Lab (test) ID, organizing results for each gene. The actual results are populated in the Observation Value (OBX-5), sourced from GT_PT_Translator.csv, while Abnormal Flags (OBX-7) indicate abnormal or critical results and are also derived from GT_PT_Translator.csv. Additional details, such as Observation Result Status (OBX-11) and timestamps, are managed within the script. The NTE segment further provides a structured way to include lab-specific comments, ensuring comprehensive data presentation.

**FIGURE 4:**
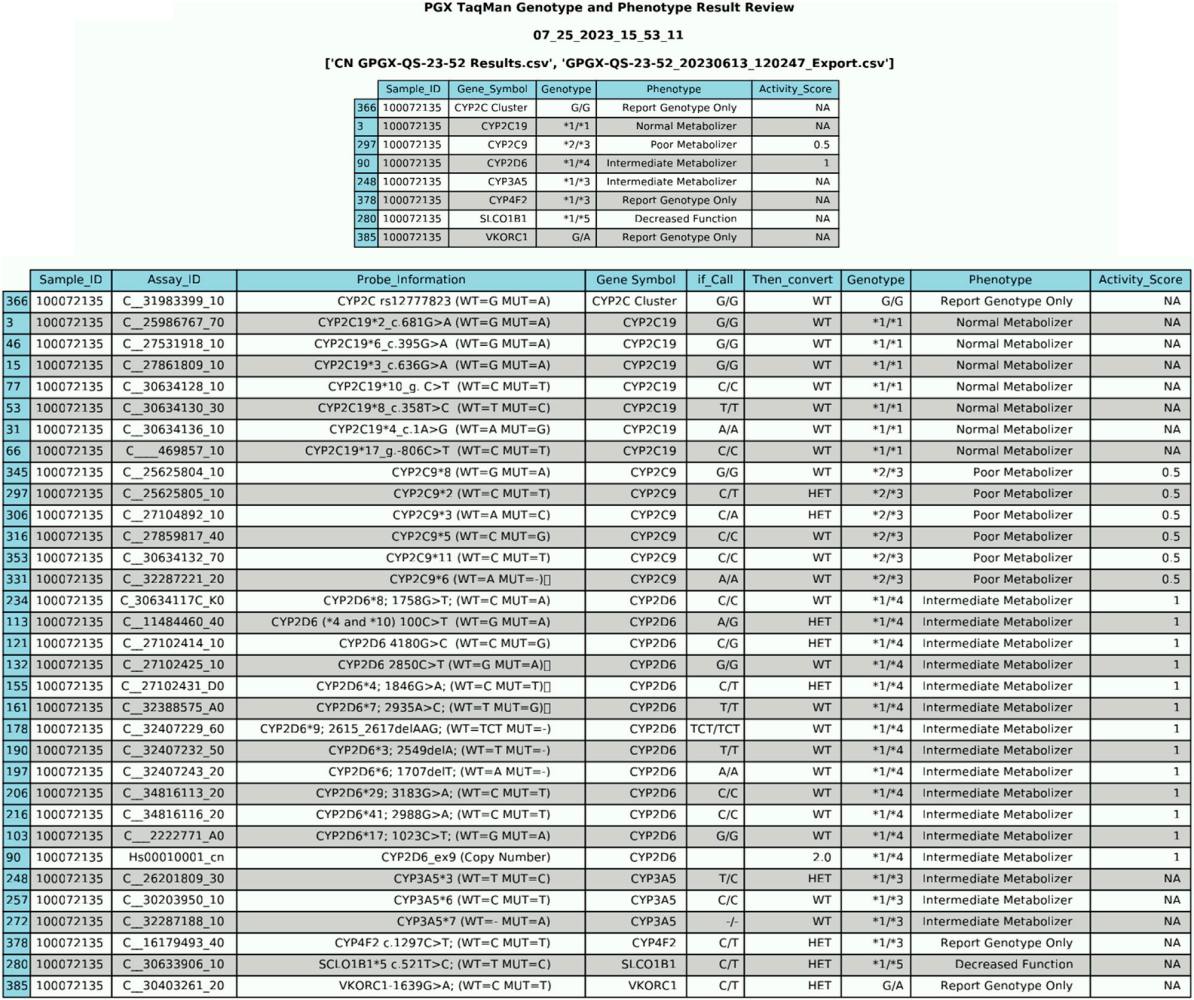
PDF Output File from Data Generation. The PDF file serves as a preliminary review of data before its integration into the Electronic Health Record (EHR). It provides a comprehensive overview of genotype/phenotype information and individual sample calls.

Once the HL7 message is constructed, the generated data is converted into two distinct output files: HL7 and PDF. The PDF file serves the purpose of internal laboratory data review/document control. As illustrated in Figure 4, this documentation includes comprehensive genotype/phenotype information along with the call for each individual sample tested allowing for easy review by the performing laboratory technologist. To ensure traceability, each PDF file includes the date of generation and the raw file names used to produce the data, facilitating a clear and transparent record of the data’s origin. This comprehensive approach not only enhances data integrity but also supports efficient clinical workflows.

**FIGURE 5:**
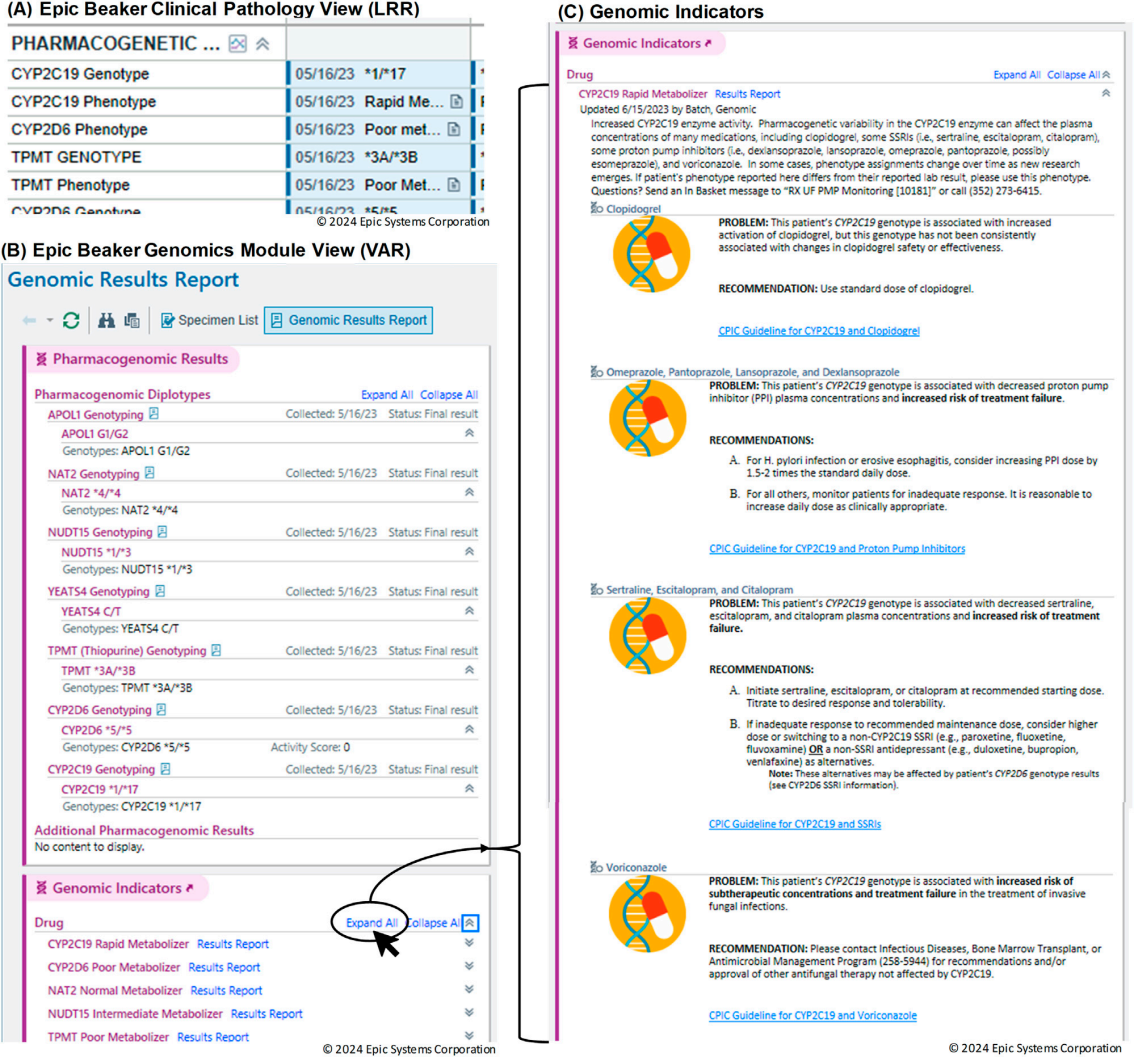
Visualization of genomic data in the Electronic Health Record (EHR). **(A)** Results from the standard Clinical Pathology (CP) Long Read Report (LRR) database displayed in the Results Review tab. **(B)** Results from the Genomics Module (Variant (VAR)). **(C)** Link to genomic indicators from the Genomics Module results view.

Visualization of the data in the EHR is shown in Figure 5. In Figure 5A, results from the standard CP (LRR) database are shown in the Results Review tab. This format lacks interactivity and does not provide genomic indicators or drug information. In contrast, the Genomics Module is designed to be more intuitive, enabling users to interact with and interpret complex genomic data more efficiently. Figure 5B presents the results from the Genomics Module (VAR), demonstrating a more sophisticated and user-friendly interface. Additionally, Figure 5C illustrates the link to genomic indicators from the Genomics Module results view. This connection allows clinicians to easily access relevant genomic information, improving the overall clarity and comprehensibility of the presented data. The PGx genomic indicator offers several notable functionalities, including interpretation language for both providers and patients, potential drug-gene interactions with recommendations, and a link to lab results. This shift to the Genomics Module offers a streamlined experience for accessing relevant genomic data within a patient’s record, making it easier for providers to review and act upon the results during clinical decision-making.

**FIGURE 6:**
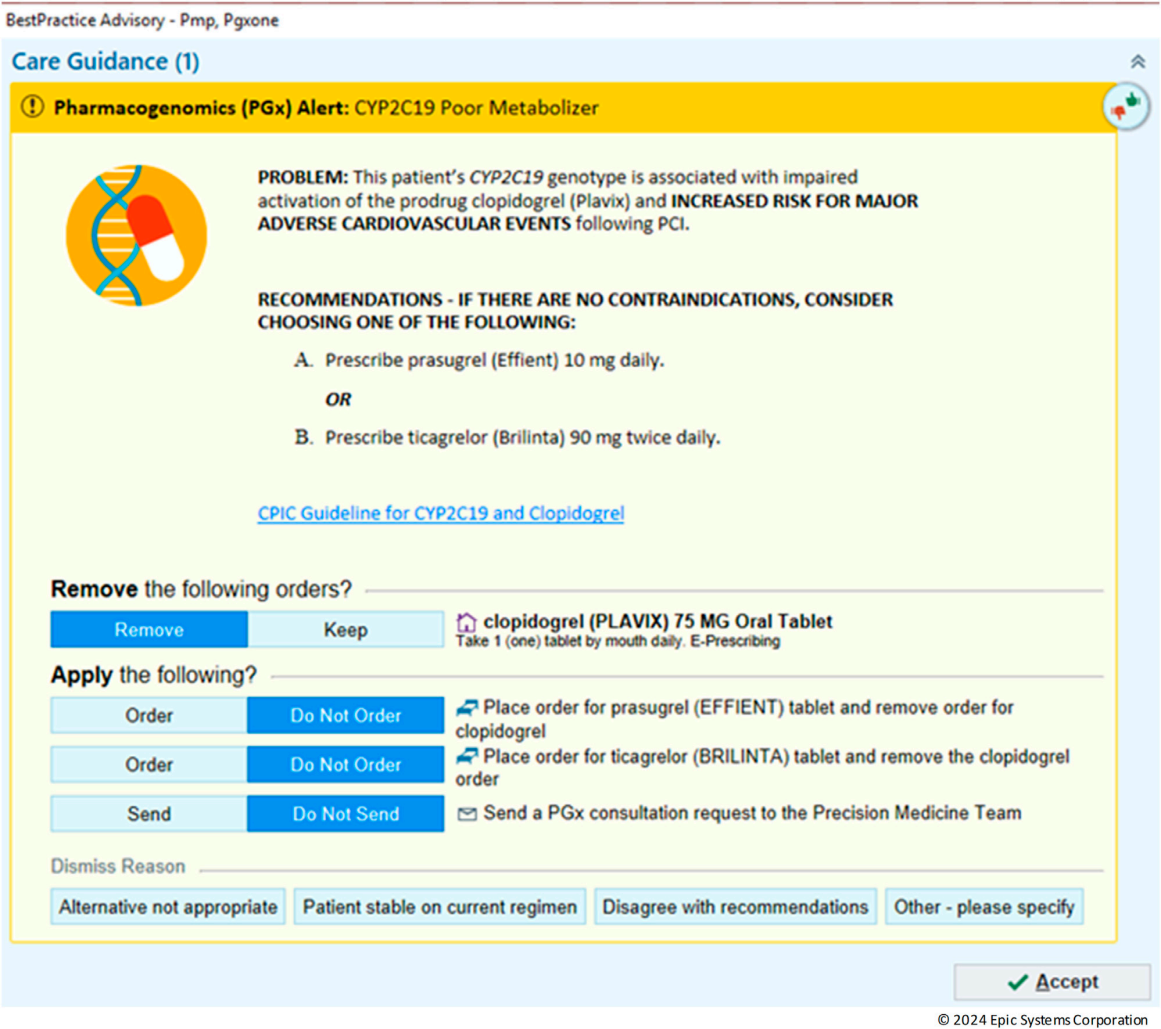
Example clinical decision support (CDS) alert.

In the realm of personalized medicine, CDS play a crucial role in guiding clinicians by providing timely notifications based on patient-specific data (Lemke et al., 2023). An example CDS is shown is Figure 6 with an alert for *CYP2C19* Poor metabolizer. In this scenario, the default is to remove the clopidogrel order however, the provider can override this by choosing to keep the order or dismiss the alert all together. With the integration of the Genomics Module into the clinical workflow, legacy CDS were revamped into a new format that uses the genomic indicators as triggers. In the past, LRR results from either genotype or phenotype were employed to trigger CDS. Although this method could meet basic requirements for constructing suitable PGx-CDS, it had several significant drawbacks. Firstly, it lacked the capability to assess complex situations such as varying activity scores (e.g., *CYP2D6* or *CYP2C9*) or updated guidelines. Secondly, maintenance was challenging, as the rule had to cover all possible genotypes for a certain phenotype. The introduction of genomic indicators as the CDS trigger criteria provided a solution to overcome these limitations. By the end of 2023, more than 11,000 patient results had been transitioned to the new VAR results. This process included the construction of 50 different indicators and the conversion of 50 CDS to the new format, using genomic indicators as triggers. When comparing 1 year before implementation to 1 year post implementation, there was a 112% increase in the number of alerts fired and interreacted with by providers (from 1,518 to 3,218).

## 4 Discussion

The successful adoption of the Genomics Module, achieved through Health Informatics and multi-team collaboration, significantly enhanced the delivery of PGx data in a clinical setting. This success is evidenced by three key measures. First, the project achieved improved integration efficiency, allowing for incorporation of PGx data and automated processes into the existing system, which reduced manual data entry and inherently enhanced data accuracy. Second, it demonstrated enhanced CDS through the complete conversion of both old and new PGx-CDS alerts to more reliable genomic criteria with the use of genomics indicators. Finally, increased user adoption was observed, reflected in the effective delivery of comprehensive PGx information to healthcare providers and a notable rise in awareness and utilization of PGx results in clinical practice. These outcomes collectively underscore the successful implementation and positive impact of the Genomics Module on PGx data management and its clinical application.

While this project is not the first to address the integration of a Genomics Module into healthcare systems, it tackles a critical challenge in genomic integration: automation and adherence to HL7 standards (Caraballo et al., 2020). Similar to the approach taken by Caraballo et al., the process implemented includes creating discrete genetic results, developing CDS rules, and integrating genomic results into the EHR workflow. However, this project goes further by providing a technical roadmap for healthcare institutions looking to implement or improve their genomic data management systems, with the aim to offer readers a comprehensive understanding of the essential technical components required for successful integration and transition. This includes an in-depth examination of data structures, and system architectures that support genomic data integration. The focus on the automation process using HL7 standards is particularly noteworthy, as it addresses the growing need for efficient and accurate handling of large-scale genomic data in clinical settings.

The utilization of HL7 standards enhances scalability and replication capabilities significantly (Hussain et al., 2018; Dolin et al., 2018). The use of health informatics principles facilitated the development of a solution that streamlines the interpretation of PGx data and generates standardized information in HL7 format compatible with the Genomics Module. Details on implementing Epic’s Genomic Module and enhancing PGx management are available as an open-source solution on GitHub (https://github.com/UF-Health-Molecular-Pathology/PGX_HL7.git). This repository contains detailed documentation and source code covering the entire integration process, including analyzing PGx TaqMan Genotyping data from Quantstudios and HL7-based communication between instruments and the EHR.

Overall, the Genomics Module significantly improves the utilization of genetic data, overcoming the limitations of traditional PDF reports (Caraballo et al., 2020). It tackles the challenge of scattered and inconsistent genomic information, enabling healthcare providers to gain a holistic understanding of all genomic results. This encompasses a wide range of results, including PGx, germline, and somatic mutations. In the realm of PGx, the Genomics Module exhibits its prowess by automatically interpreting results and incorporating indicators into the patient’s chart. This feature empowers healthcare providers by offering them a clear understanding of the interpretations and enabling them to receive genomics-guided recommendations. This integration not only streamlines the process of interpreting complex genetic data but also enhances the precision and personalization of healthcare. Providing a comprehensive view of a patient’s genomic information allows for more informed decision-making and contributes to the advancement of personalized medicine. Ultimately, the Genomics Module serves as a powerful tool in the hands of healthcare providers, paving the way for a future where genetic data plays a pivotal role in patient care (Lau-Min et al., 2021).

## 5 Limitations

The implementation of precision medicine care using the Genomics Module encountered several challenges: (1) While the HL7 standard aids in integrating genomics results, incorporating data from third-party instruments that lack direct interface capabilities with Epic (e.g., using middleware like Data Innovations) involves a complex process. If this integration work cannot be completed, manual input of results and genomic indicators may still be required to fully utilize the capabilities of the Genomics Module. (2) The ability to create rules for interpreting activity scores derived from genotype is currently limited to the UFHPL. While genomic indicators are used to report and interpret these scores, they cannot directly translate from alleles or duplications. However, with the upcoming Epic update, healthcare providers and IT teams will gain the capability to create their own rules based on activity scores, extending the customization of these interpretations beyond the laboratory. (3) The current presentation of Genomic Indicators is not ideal for illustrating complex gene-drug interactions involving multiple genetic variants. For instance, medications like sertraline, which is influenced by both *CYP2C19* and *CYP2B6* mutations, have their relevant information scattered across different genetic indicators. This layout forces users to navigate through multiple sections to gather comprehensive data. Similarly, thiopurine medications (such as thioguanine, azathioprine, and mercaptopurine) are significantly impacted by both *TPMT* and *NUDT15* genes, yet their information is not consolidated. To address this limitation, a more effective approach could involve organizing indicators by medication rather than by gene. Alternatively, implementing dynamic alerts capable of assessing multiple drug-gene interactions simultaneously could enhance the system’s utility and user experience. (4) The module does not fully address the issue of phenoconversion, which can occur when drug-drug interactions inhibit or induce *CYP* enzyme activity, potentially altering a patient’s phenotype. This limitation stems from the reliability of active medication information in patient profiles rather than the Genomics Module itself. Currently, patient-specific consultation notes are the primary method to address potential phenoconversion. These limitations highlight areas for future improvement to enhance the Genomics Module’s effectiveness in supporting precision medicine care, including better integration of third-party results, improved result categorization, expanded activity score reporting capabilities, optimized display of gene-drug interactions, and more comprehensive handling of phenoconversion scenarios.

## Data Availability

The original contributions presented in the study are included in the article/supplementary material, further inquiries can be directed to the corresponding author.
